# A Rare Case of a Synchronous Anaplastic Carcinoma Thyroid with Ductal Carcinoma Breast

**DOI:** 10.1155/2014/468159

**Published:** 2014-04-13

**Authors:** Saptarshi Ghosh, P. B. Ananda Rao, Shreyasee Sarkar, Sivasankar Kotne, S. P. V. Turlapati, Anindita Mishra

**Affiliations:** ^1^Department of Radiotherapy, GSL Medical College & General Hospital, Rajahmundry 533294, India; ^2^Department of Pathology, GSL Medical College & General Hospital, Rajahmundry 533294, India; ^3^Department of Radiology, GSL Medical College & General Hospital, Rajahmundry 533294, India

## Abstract

Dual malignancy was first reported by Billroth in 1889. Incidence of second malignancy in cancer patients is as high as 10%, but synchronous anaplastic thyroid cancer along with breast tumor is a rare entity. We present a case of a 61-year-old female with a synchronous anaplastic carcinoma thyroid with ductal carcinoma breast. The plausible association of breast cancers with thyroid carcinomas should thus be evaluated in larger cohort studies. More importantly, this report is to highlight the unusual synchronous occurrence of anaplastic thyroid cancer with ductal breast cancer and the therapeutic challenges involved in such cases.

## 1. Introduction

Dual malignancy was first reported by Billroth in 1889 [1]. The second malignancy can be synchronous or metachronous. Synchronous malignancy is two or more histologically different malignancies detected within six months of the diagnosis of the first primary malignancy. Certain criteria have been laid down to diagnose synchronous malignancy; for example, both the tumors should be malignant and neither should be a metastasis from the other, and the tumors should be separate from each other in terms of microscopic and morphologic features [1]. Though the incidence of a second primary tumor in cancer patients is about 10% [2], the synchronous occurrence of anaplastic thyroid cancer with ductal cell carcinoma of the breast is an extremely rare entity and is the first case reported in India. We report a rare case of a 61-year-old female who presented with anaplastic thyroid cancer after hemithyroidectomy (residual disease) with right lung metastasis along with left ductal cell carcinoma breast. The occurrence of synchronous breast malignancy along with thyroid cancers may be linked genetically or hormonally as both are more common in postmenopausal women [3].

## 2. Case History

**Figure 1:**
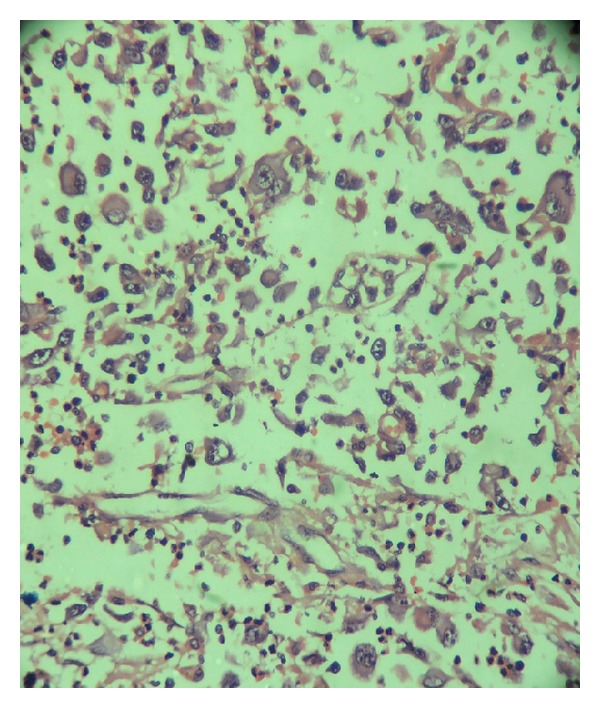
The tumor was composed of large sized, bizarre shaped cells with ample dense eosinophilic cytoplasm and highly pleomorphic vesicular nuclei with prominent nucleoli, without showing any organoid pattern. Multiple mitotic figures and neutrophilic infiltrate are also seen (H&E stain 100x).

**Figure 2:**
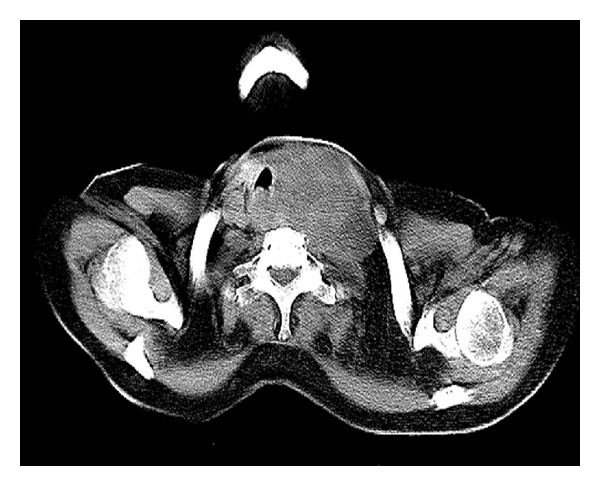
CECT neck showing a 5.9 cm × 4.3 cm lesion involving the left lobe of thyroid and isthmus encasing the left common carotid artery and subclavian artery and extending into the left carotid space and anterosuperior mediastinum.

**Figure 3:**
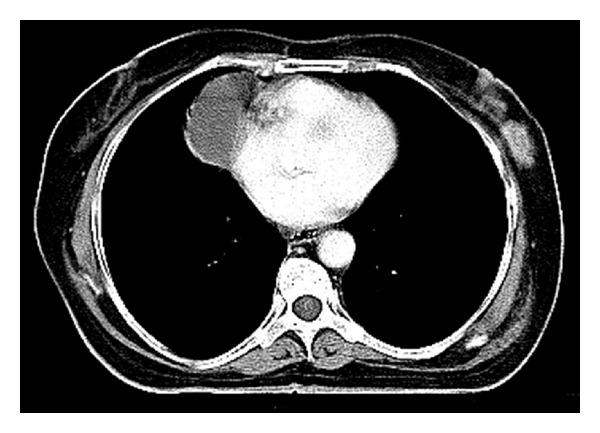
A well-defined lesion of size 4.1 cm × 4.6 cm in the middle lobe of right lung abutting the mediastinal pleura along with left axillary lymphadenopathy.

**Figure 4:**
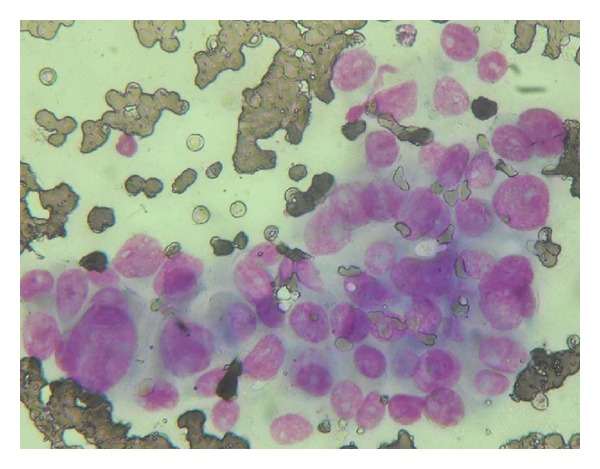
Smear shows malignant ductal epithelial cells arranged in sheets discretely. The cells are large showing pleomorphic nuclei with prominent nucleoli and ample dense greyish cytoplasm (H&E stain 400x).

A 61-year-old female presented with left anterior neck swelling after hemithyroidectomy with dyspnea and stridor. The neck swelling was there for the last 5 years, without any change in size. For the last 3 months, it suddenly increased diffusely up to a size of 6.0 cms × 3.5 cms. She underwent hemithyroidectomy for the thyroid swelling elsewhere. Histopathological examination of the neck swelling confirmed the diagnosis of anaplastic thyroid cancer (Figure 1). Contrast enhanced CT scan of the neck showed well-defined, moderately enhancing lesion of 5.9 cm × 4.3 cm size involving the left lobe of thyroid and isthmus encasing the left common carotid artery and subclavian artery and extending into the left carotid space and anterosuperior mediastinum (Figure 2). It also revealed a well-defined lesion of size 4.1 cm × 4.6 cm in the right middle lobe of lung abutting the mediastinal pleura and left axillary lymphadenopathy (Figure 3). The lesion in the right lung was cytologically consistent with the diagnosis of anaplastic carcinomatous deposits. Incidentally a hard nodular lump of size 3.0 cm × 3.5 cm was found in the upper outer quadrant of the left breast. Fine needle aspiration cytology of the left breast lump (Figure 4) and the left axillary lymph node was consistent with the diagnosis of ductal cell carcinoma of the left breast. Patient was started on palliative chemotherapy in the intensive care unit with supplemental oxygen. Chemotherapy was given with Doxorubicin and Cisplatin. Patient responded well to the treatment and the neck mass size reduced, thereby subsiding her dyspnea and stridor. Palliative radiation to the neck was planned.

## 3. Conclusion

Anaplastic thyroid cancer is a rare malignancy comprising of 1-2% of all thyroid cancers [4, 5]. Nevertheless it has a dismal prognosis with a median life expectancy of about 4 months [4, 6]. The poor prognostic signatures in our patient here were age >60 years, presence of lung metastasis, R2 resection, multiple lymph nodal involvement, presence of acute symptoms like dyspnea and stridor, patient unfit for adjuvant radiation, and presence of a synchronous cT2 N1 M0 left breast cancer. Doxorubicin is a drug which acts in both anaplastic thyroid cancer and ductal carcinoma breast. The response rate of Doxorubicin in anaplastic thyroid cancer is 22% [5]. The poor cardiorespiratory condition of the patient eluded the chance of a tracheostomy in this patient.

The synchronous occurrence of anaplastic carcinoma thyroid and ductal carcinoma breast remains a therapeutic challenge especially when the patient presents with acute symptoms. Synchronous thyroid and breast malignancies are often attributed to genetic aberrations and hormonal influences.

Thyroid and breast are both under the influence of same hormones. While estrogen plays a role in the development of the thyroid gland, TSH receptors are found to be present in breast tissue. Also increased thyroid peroxidase levels have been associated with better prognosis in breast cancers [7]. Data from the Connecticut Tumor Registry demonstrated a significantly elevated risk of developing thyroid cancer following breast cancer and vice versa [8].

Both these cancers can also be genetically correlated with p53 or PTEN mutations, which is an extremely rare situation. Silencing of tumor suppressor gene PTEN has been found in anaplastic thyroid cancer. PTEN has also been found to be mutated or deleted or silenced in sporadic breast cancers [9].

Progression from papillary thyroid cancer to anaplastic carcinoma thyroid could be favored by TP53 mutations [10]. p53 mutations are seen in breast cancers and have been seen to affect its prognosis with positive p53 mutations having a worse prognosis.

So the family members of these patients should be advised to undergo genetic screening at higher centers to exclude any probability of a genetic aberration which could link these two synchronous cancers and also a larger cohort study is needed to validate the synchronous presence of thyroid and breast malignancies and the genetic and hormonal factors associated with them.
